# Interferon-alpha and MxA inhibit BK polyomavirus replication by interaction with polyomavirus large T antigen

**DOI:** 10.1016/j.bj.2023.100682

**Published:** 2023-12-07

**Authors:** Hsin-Hsu Wu, Yi-Jung Li, Cheng-Hao Weng, Hsiang-Hao Hsu, Ming-Yang Chang, Huang-Yu Yang, Chih-Wei Yang, Ya-Chung Tian

**Affiliations:** a Kidney Research Center, Department of Nephrology, Linkou Chang Gung Memorial Hospital, Taoyuan, Taiwan; b Department of Medicine, Chang Gung University, Taoyuan, Taiwan; c Graduate Institute of Clinical Medical Sciences, College of Medicine, Chang Gung University, Taiwan

**Keywords:** BK polyomavirus, Kidney transplant, Interferon, MxA

## Abstract

**Introduction:**

BK Polyomavirus (BKPyV) infection is a common complication in kidney transplant recipients and can result in poor outcomes and graft failure. Currently, there is no known effective antiviral agent. This study investigated the possible antiviral effects of Interferon alpha (IFNα) and its induced protein, MxA, against BKPyV.

**Methods:**

*In vitro* cell culture experiments were conducted using human primary renal proximal tubular epithelial cells (HRPTECs). We also did animal studies using Balb/c mice with unilateral kidney ischemic reperfusion injury.

**Results:**

Our results demonstrated that IFNα effectively inhibited BKPyV *in vitro* and murine polyomavirus in animal models. Additionally, IFNα and MxA were found to suppress BKPyV TAg and VP1 production. Silencing MxA attenuated the antiviral efficacy of IFNα. We observed that MxA interacted with BKPyV TAg, causing it to remain in the cytosol and preventing its nuclear translocation. To determine MxA's essential domain for its antiviral activities, different mutant MxA constructs were generated. The MxA mutant K83A retained its interaction with BKPyV TAg, and its antiviral effects were intact. The MxA T103A mutant, on the other hand, abolished GTPase activity, lost its protein-protein interaction with BKPyV TAg, and lost its antiviral effect.

**Conclusion:**

IFNα and its downstream protein, MxA, have potent antiviral properties against BKPyV. Furthermore, our findings indicate that the interaction between MxA and BKVPyV TAg plays a crucial role in determining the anti-BKPyV effects of MxA.

## Highlights

• BK Polyomavirus (BKPyV) infection is common in kidney transplant and can result in graft failure.
• There is no effective antiviral agents against BKPyV currently.
• This study demonstrates Interferon alpha and MxA reduce BKPyV replication.
• MxA interacts with viral protein TAg, and prevents its nuclear translocation.
• T103A MxA loses its interaction with TAg and is a dominant negative mutant.

## 1 Introduction

Kidney transplantation is the best treatment for end-stage renal disease with good quality of life and survival outcomes [1,2]. However, kidney transplant recipients require lifelong immunosuppressants, compromising their immune system and making them vulnerable to infections. One common complication is human polyomavirus BK (BKPyV) infection. BKPyV infects nearly 80 % of the human population and causes lifelong persistence within epithelial cells of the urinary tract [3]. BKPyV infection remains asymptomatic in most people throughout their lifetime. However, immunocompromised individuals are susceptible to BKPyV-associated diseases. The prevalence of viruria in kidney transplant recipients is about 30%–50 %; among these affected individuals, 30%–40 % of them have viremia [4]. The prevalence of BKPyV-associated nephropathy (BKPyVAN) is about 20%–40 % among the viremic patients [[5], [6], [7]]. Recent studies showed the kidney graft failure rate due to BKPyVAN is between 5 % and 50 % according to the histology severities [5,8].

Since there are currently no effective antiviral treatments for BKPyV infection, the most common strategy to treat BKPyVAN is to decrease the patient's immunosuppressants. However, this may increase the risk of graft rejection. Therefore, finding an effective treatment for BKPyVAN is vital in renal transplant recipients.

Interferons have been discovered for nearly half a century and are regarded as antiviral agents which interfere with virus replication [9]. Virus-infected cells can secrete interferons, which promote cytokines release and enhance natural killer cells' function [10]. Type I interferons (IFNs), including IFNα, IFNβ, and IFNω. Type II IFN is mainly IFNγ [11]. These IFNs mediate various biological activities, including antiviral activity, cell growth, differentiation, apoptosis, and immune responses [12]. IFNα/β binds to a common heterodimeric receptor composed of IFNα/β receptor 1 (IFNAR1) and IFNα/β receptor 2 (IFNAR2) and then activates the Janus kinase (JAK) family and signal transducers and activators of the transcription (STATs) family [13]. Phosphorylation of STAT1 by ERK1/2 and p38 MAPK facilitates the interaction of STAT1 with the basal transcription machinery, enabling full expression of antiviral genes. These IFN-stimulated genes (ISGs) include protein kinase R (PKR), 2′,5′-oligoadenylate synthetase (OAS), ISG15, and Myxovirus resistant (Mx) gene [14].

The human myxovirus resistance protein A (MxA) is one of the IFN-stimulated gene products. MxA is a dynamin-like GTPase. It has a wide range of antiviral activity against various DNA and RNA viruses. MxA inhibits influenza A virus replication by blocking endocytic traffic of incoming virus particles and decreases nucleoprotein synthesis [[15], [16], [17]]. It also inhibits hepatitis B virus replication through binding and inactivation of their ribonucleocapsid [18]. Since there is no effective treatment for BKPyV infection, and the antiviral effects of IFN and MxA on BKPyV remain unclear, it is crucial to study the possible anti-BKPyV role of MxA and its underlying mechanism.

In this study, we investigated the antiviral abilities against BKPyV of type I Interferon and its downstream product, MxA. In addition, we also studied the anti-BKPyV mechanism of MxA by studying the antiviral effects of different function defect MxA mutants and the possible protein-protein interaction between BKPyV viral protein and MxA.

## 2 Materials and methods

### 2.1 Cell culture

Human primary renal proximal tubular epithelial cells (HRPTECs) were purchased from the American Type Culture Collection (ATCC). Cells were cultured in DMEM/F-12 1:1 (Gibco, NY, USA) containing 1 % fetal bovine serum under 37 °C and 5 % CO2. Human and mouse recombinant IFNα were purchased from R&D Systems (MN, USA).

For gene transfection experiments, cells were incubated with 1:1 plasmid and X-tremeGene from Roche (Basel, Switzerland) for 2 h at 4 °C and then washed with fresh culture medium before further experiments. For gene knockdown experiments, cells were incubated with 1:2 of siRNA or scrambled control RNA and Dharmafect from Horizon Discovery (Cambridge, UK) for 12 h and washed with fresh culture medium before further experiments. For cell culture experiments, at least three independent experiments were performed.

### 2.2 *In vivo* experiment

Balb/c female mice at the age of 8–10 weeks old were used. Mice were anesthetized by intraperitoneal injection (i.p.) of 65 mg/kg pentobarbital. The renal artery was then identified, and we performed unilateral kidney ischemic reperfusion injury (IRI) by clamping the renal artery for 30 min. After unilateral kidney IRI, 2 x 10^6^ plaque-forming units of murine polyomavirus (MuPyV) wild-type A2 strain were inoculated i.p. These mice were treated with either PBS or murine IFN α 1 M IU/kg subcutaneously. After 7 days of inoculation, the mice were sacrificed, and mice kidneys were harvested and homogenized using a rotor-stator homogenizer. RNA was extracted from the kidneys using commercial RNA extraction kits (RNeasy kits, Qiagen, Hilden, Germany). Reverse transcription quantitative polymerase chain reaction (RT-qPCR) was used to measure MuPyV viral protein VP1 mRNA expression in the kidneys. RNase-free DNase I was used to remove DNA contamination in our RNA samples prior the RT-qPCR. A minus reverse transcriptase control was used to track genomic DNA contamination. For animal studies, each experimental group comprised 4 to 6 animals.

### 2.3 Quantitative measurement of BKPyV and murine polyomavirus (MuPyV) load

BKPyV load in the culture medium or cell lysate was determined by qPCR as previously described [19]. DNA was extracted from the specimens using a QIAampDNA Mini Kit (Qiagen, Hilden, Germany). Primers of BKPyV TAg, forward: 5′-CTG TCC CTA AAA CCC TGC AA-3′ and reverse: 5′-GCC TTT CCTTCC ATT CAA CA-3’.

Semi-quantitative MuPyV viral load was reported as fold changes in viral VP1 mRNA relative expression using 18S ribosomal RNA (18S) as reference. The 2-ΔΔCt method was used to calculate fold changes. Primers of MuPyV VP1, forward primer: 5′-TGGGAGGCAGTCTCAGTGAAA-3′, and reverse primer, 5′-TGAACCCATGCACATCTAACAGT-3′. Two technical replicates of each PCR reaction were performed.

### 2.4 cDNA constructs

We used standard molecular biology techniques for DNA isolation, analysis, and cloning [20,21]. MxA cDNAs were cloned into mammalian cell expression vectors pcDNA3.1 (Invitrogen). Three MxA mutants were created to study the underlying antiviral mechanism against BKPyV, including two GTP binding mutants, K83A and D250 N, and one GTPase activity-abolished mutant, T103A. Mutations were introduced into the MxA cDNA by PCR-based site-directed mutagenesis. The sense primers: K83A, 5′-GACCAGAGCTCGGGCGCTAGCTCC-3′; T103A, 5′-AGCGGGATCGTGGCCAGATGCCCGCTG-3′ [22]; D250 N, 5′ CTTGACGAAGCCTAATCTGGTGGACAAAGG 3′ and their complementary antisense primers. The resulting PCR products were sequenced to confirm the mutations. We introduced the FLAG epitope (DYKD-DDDK) at the N terminus of wild-type MxA and MxA mutants to create FLAG-tagged plasmids.

### 2.5 Western blot analysis

We performed Western blot analysis as previously described [19]. Since there is no commercially available anti-BKPyV TAg antibody, we used an anti-SV40 TAg antibody (Calbiochem, CA, USA) due to its cross-reactivity. Anti-VP1 antibody was purchased from Abnova (Taipei, Taiwan). Anti-MxA antibody and anti-GAPDH antibody were purchased from Abcam (Oregon, USA). Ant-FLAG antibody was purchased from Invitrogen (USA).

### 2.6 Co-immunoprecipitation and Immunoblot analysis

HRPTECs were transfected with TAg plasmid and FLAG-tagged wild-type (WT) MxA or mutants MxA plasmids using the X-tremeGene from Roche (Basel, Switzerland). After 12 h of transfection, co-immunoprecipitation analysis was performed. HRPTECs were lysed in 50 mm Tris (pH 8.0), 150 mm NaCl, 1 mm EDTA, and 0.5 % Nonidet P-40. Protein G agarose immunoprecipitation kit was used (Roche). The washed precipitates, as well as the whole cell lysates to control protein expression, were subjected to standard Western blot analysis using antibodies against FLAG, MxA, LT, and GAPDH.

### 2.7 Immunofluorescent study

HRPTECs were transfected with WT MxA plasmid for 12 h or treated with IFNα. These cells were inoculated with BKpyV 1 x 10^6^/ml. After 72 h of infection, cells were fixed with 3 % paraformaldehyde and permeabilized with 0.5 % Triton-100 in phosphate-buffered saline (PBS). MxA was labeled with anti-MxA and a green fluorescent (Alexa Fluor 488) secondary antibody (Invitrogen), and BKV TAg was labeled with anti-SV40 TAg and a red fluorescent (Alexa Fluor 647) secondary antibody (Invitrogen). The cell nucleus was stained with DAPI (4′,6-diamidino-2-phenylindole) (Invitrogen).

### 2.8 Statistical analysis

Data were presented as mean ± standard error of the mean. Student's t-test or ANOVA test with multiple comparisons were used for data comparison. A value of *p* < 0.05 was considered statistically significant.

## 3 Results

### 3.1 Interferon α inhibits BKPyV replication

**Fig. 1:**
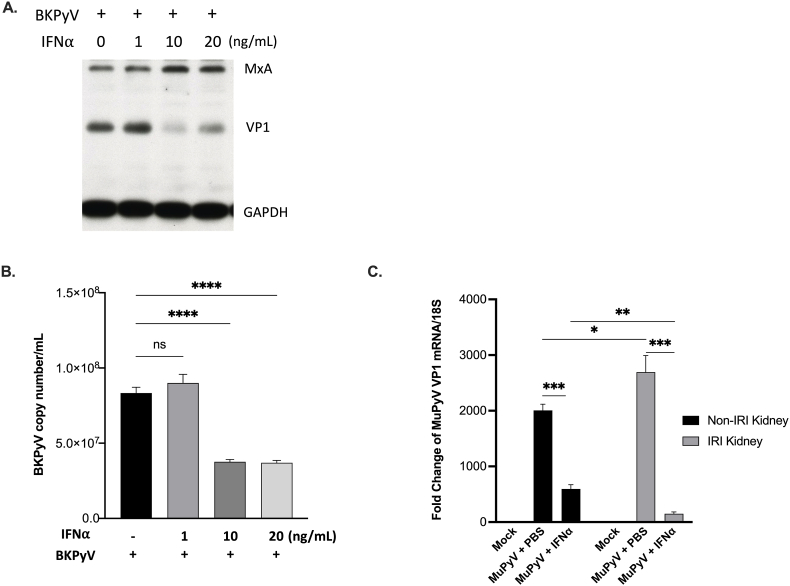
**Inhibition of BKPyV replication by IFNα** HRPTECs were infected with BKPyV (1 × 10^6^ copies/mL) for 2 h and then incubated in the presence or absence of human IFNα (0−20 ng/mL) for an additional 72 h. At the end of experiments, cell lysates were collected for Western blot analysis (A) to determine the protein expression of MxA and VP1, and the BKPyV viral titer was measured by qPCR (B) from the collected supernatant. (C) Eight-to ten-week Balb/c female mice received 30 min-ischemic reperfusion injury (IRI) of a unilateral kidney. Mice were then intraperitoneally inoculated with MuPyV wild-type strain (2 × 10^6^ plaque-forming units). Two doses of IFNα (1 MIU/Kg) or PBS were subcutaneously injected on day 1 and day 4. After seven days of inoculation, the mice were sacrificed, and fold change of MuPyV VP1 mRNA relative expression using 18S rRNA (18S) as reference in the IRI and non-IRI kidneys was determined by RT-qPCR. The results of cell culture experiments represent three independent experiments. For animal study, each experimental group comprised 4 to 6 animals. The results are presented as mean ± SEM. Statistical significance: ns, not significant; ∗, *p* < 0.05; ∗∗, *p* < 0.01; ∗∗∗, *p* < 0.005; ∗∗∗∗, *p* < 0.001.

To assess the potential inhibitory effect of IFNα on BKPyV replication, we employed *in vitro* and *in vivo* experimental models to evaluate its antiviral activity. HRPTECs were infected with BKPyV and subsequently treated with various doses of human IFNα for 72 h. Cell lysates were collected for Western blot analysis, and the culture media were collected to determine the viral load of BKPyV. The higher dose of IFNα resulted in more expression of MxA and reduced viral protein VP1 expression [Fig. 1A]. The results of qPCR revealed a significant reduction in BKPyV viral load when cells were stimulated with 10 ng/mL of IFNα compared to those without IFNα treatment (8.33 ± 0.67 × 10^7^ versus 3.77 ± 0.25 × 10^7^ copies/mL) [Fig. 1B]. However, there was no further decrement in BKPyV viral load when cells were treated with 20 ng/mL of IFNα.

To assess the inhibitory effect of IFNα on viral replication *in vivo*, we initially inoculated BKPyV intraperitoneally into Balb/c mice. Despite the detection of BKPyV in the urine on Day 4, the virus was rapidly eliminated and became undetectable by Day 14 (data not shown). Consequently, we employed murine polyomavirus (MuPyV) as a surrogate for human polyomavirus in our subsequent *in vivo* investigation. To mimic ischemia-reperfusion injury (IRI) in kidney transplantation, we utilized a unilateral renal artery IRI model. Following a 30-min ischemic period of the unilateral renal artery, Balb/c mice were intraperitoneally inoculated with MuPyV. Our experiment showed the highest MuPyV VP1 mRNA expression occurred at day 7 post inoculation, and the viruses were nearly totally cleared at day 28 [supplement figure]. Using this polyomavirus infection animal model, we applied two doses of IFNα (1 MIU/Kg) or PBS subcutaneously administrated on Day 1 and Day 4. On day 7, the mice were sacrificed, and RT-qPCR was employed to determine the expression of MuPyV VP1 mRNA in both the IRI kidneys and the non-IRI kidneys. [Fig. 1C] illustrates significantly elevated MuPyV VP1 mRNA expression in the IRI kidneys when compared to the non-IRI kidneys. Notably, the mice treated with IFNα exhibited a significant reduction in VP1 mRNA expression compared to those receiving PBS injection [Fig. 1C].

### 3.2 MxA has antiviral activity against BKPyV

**Fig. 2:**
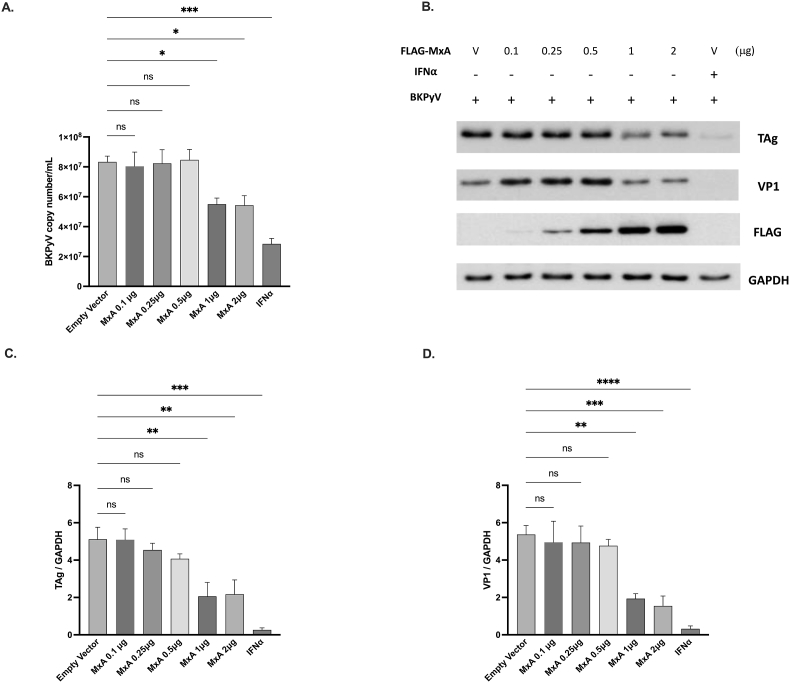
**Inhibition of BKPyV replication by MxA** HRPTECs were transfected with varying doses (0.1a2 μg) of FLAG-tagged MxA-expressing plasmids or an empty plasmid for 12 h. Subsequently, they were infected with BKPyV (1 × 10^6^ copies/mL) for 2 h. After replacing the medium, the cells were cultured for an additional 72 h. BKPyV-infected cells transfected with the empty vector served as the negative control, while BKPyV-infected cells treated with 10 ng/mL of IFNα served as the positive control. At the end of the experiments, viral titer analysis (A) was performed on the collected supernatant, and Western blot analysis (B) was conducted on cell lysates to determine the expressions of TAg, VP1, and MxA proteins. Bar graphs depict the normalized levels of BKPyV TAg (C) and VP1 (D) relative to GAPDH. The data represent three independent experiments. The results are presented as mean ± SEM. Statistical significance: ns, not significant; ∗∗, *p* < 0.01; ∗∗∗, *p* < 0.005; ∗∗∗∗, *p* < 0.001.

MxA is a crucial mediator of the IFNα-mediated antiviral activity [23]. To assess whether MxA has antiviral activity against BKPyV, HRPTECs were transfected with different doses of MxA-expressing plasmids for 12 h, followed by infection of cells with BKPyV for an additional 72 h. BKPyV-infected cells treated with 10 ng/mL of IFNα were used as a positive control. Cell lysates were collected for Western blot analysis, and the supernatant of the cell culture was collected for viral titer measurement. BKPyV viral loads were significantly suppressed by higher doses of MxA-expressing plasmid transfection [Fig. 2A]. The result of Western blot analysis demonstrated that the expression of TAg and VP1 proteins was remarkably suppressed by IFNα treatment [Fig. 2B]. The expression of TAg and VP1 was significantly decreased in a dose-dependent manner in cells transfected with the MxA-expressing plasmid compared to cells transfected with the empty vector [Fig. 2C and D].

### 3.3 Silencing MxA attenuates the antiviral efficacy of interferon alpha

**Fig. 3:**
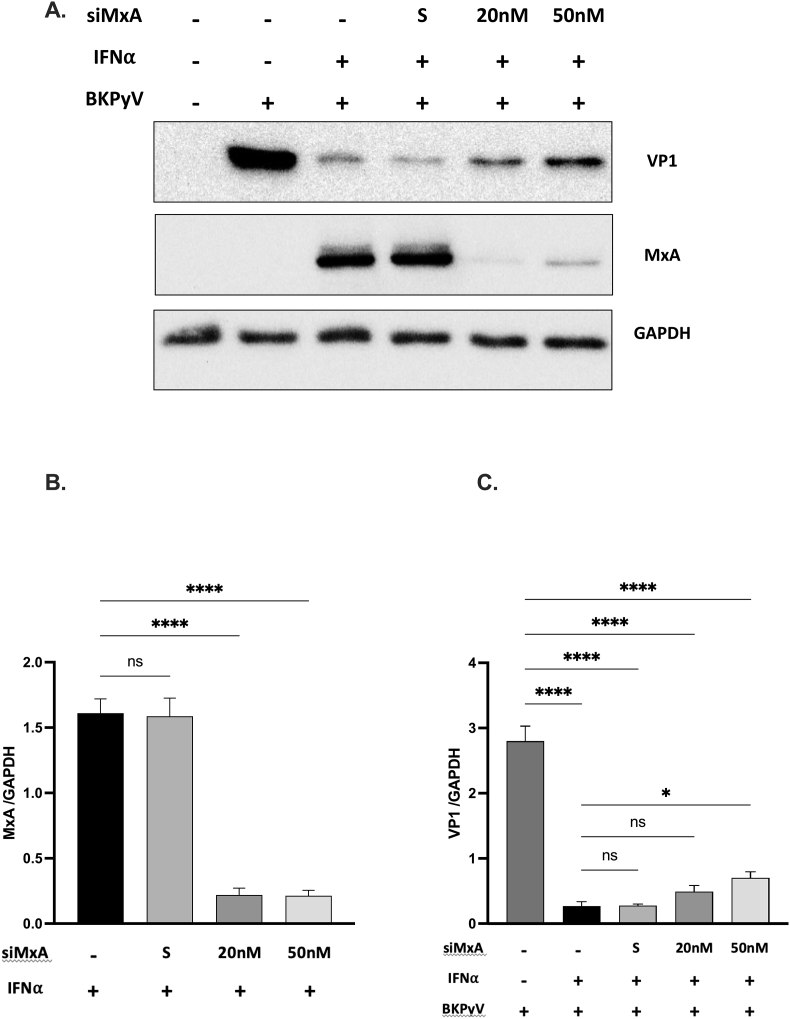
**MxA silencing diminished IFNα′s anti-BKPyV effect** HRPTECs were transfected with varying doses of MxA siRNA or scrambled RNA for 12 h. Afterward, they were treated with IFNα (10 ng/mL) and infected with BKPyV (1 × 10^6^ copies/mL) for an additional 72 h. Western blot analysis was performed to assess the expression levels of TAg, VP1, and MxA proteins (A). Bar graphs depict the normalized levels of BKPyV MxA (B) and VP1 (C) relative to GAPDH. The data represent three independent experiments. The results are presented as mean ± SEM. Statistical significance: ns, not significant; ∗, *p* < 0.05; ∗∗∗∗, *p* < 0.001.

To assess the impact of MxA silencing on the antiviral activity of IFNα, HRPTECs were transfected with MxA siRNA for 12 h to suppress MxA expression. Subsequently, cells were treated with IFNα (10 ng/mL) and infected with BKPyV for an additional 72 h. Western blot analysis revealed a significant decrease in MxA expression following siRNA transfection [Fig. 3A&3B]. The inhibitory effect of IFNα on VP1 protein expression was partially reversed upon MxA silencing [Fig. 3A and C]. These findings suggest that MxA silencing weakens the antiviral efficacy of IFNα, although its effect is not entirely abrogated, indicating the involvement of other IFNα-induced mediators.

### 3.4 GTPase activity of MxA at position 103 is crucial for its antiviral effect against BKPyV

**Fig. 4:**
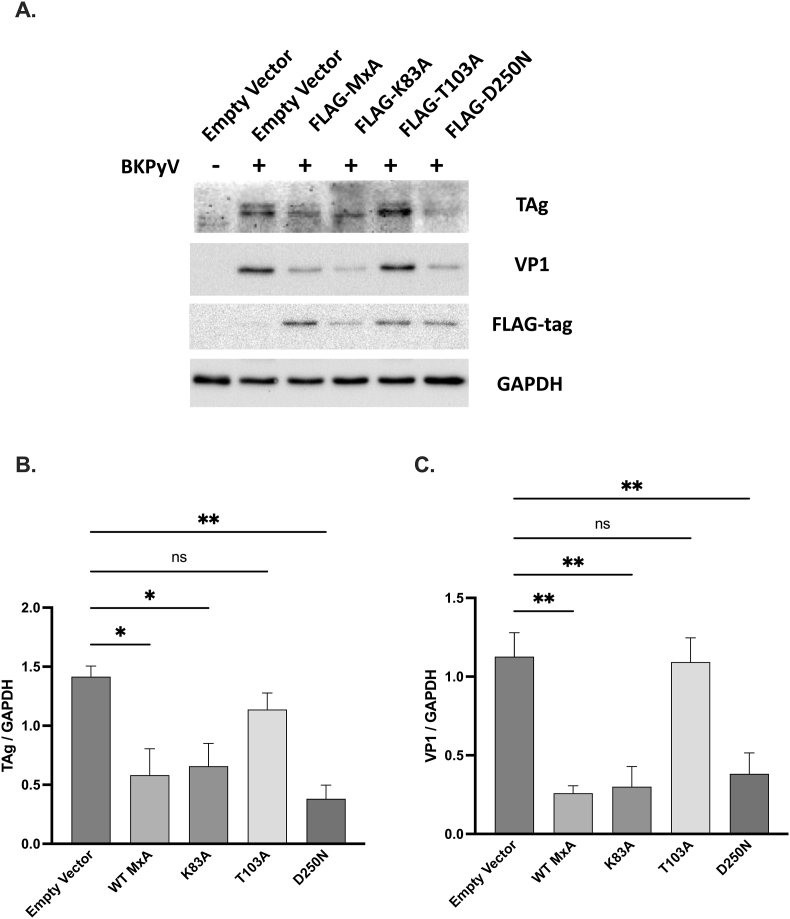
**Mutation of MxA residue 103 decreases antiviral activity against BK polyomavirus**. HRPTECs were transfected with plasmids encoding FLAG-tagged wild-type MxA, GTP binding-defective MxA mutants (K83A and D250 N), or the GTPase-deficient MxA mutant (T103A) for 12 h. Cells transfected with the empty vector served as the negative control. Subsequently, the cells were infected with BKPyV (1 × 10^6^ copies/mL) for an additional 72 h. Western blot analysis was conducted to determine the expression levels of wild-type MxA, MxA mutants, BKPyV TAg, and VP1 proteins. Bar graphs represent the normalized levels of BKPyV TAg (B) and VP1 (C) relative to GAPDH. The results are presented as mean ± SEM. Statistical significance: ns, not significant; ∗, *p* < 0.05; ∗∗, *p* < 0.01.

To determine MxA's essential domain for antiviral activities, we generated various FLAG-tagged wild-type and mutant plasmids: two GTP-binding defective mutants, K83A and D250 N, and one GTPase-deficient mutant, T103A. HRPTECs were transfected with these plasmids for 12 h and then infected with BKPyV for an additional 72 h. Western blot analysis showed that wild-type MxA plasmid transfection inhibited BKPyV TAg and VP1 expressions compared to the empty vector [Fig. 4A]. Transfection with K83A and D250 N mutants suppressed TAg and VP1 expressions, while T103A mutant transfection failed to suppress TAg [Fig. 4A and B] and VP1 protein production [Fig. 4A and C].

### 3.5 The interaction between MxA and BKPyV TAg is crucial for TAg nuclear localization

**Fig. 5:**
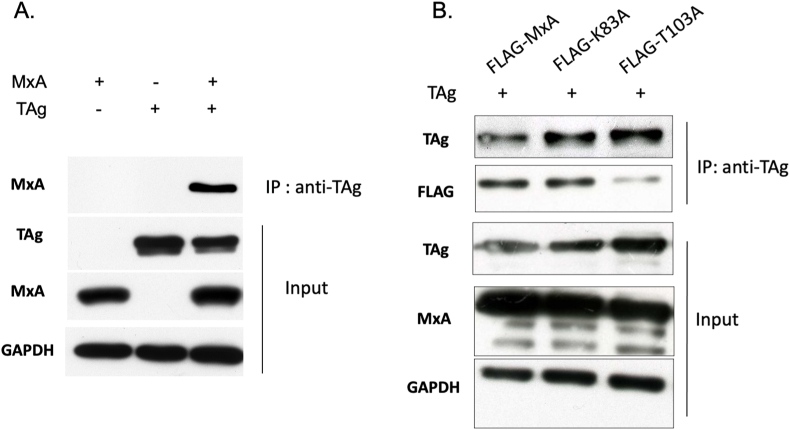
**BKPyV TAg is co-immunoprecipitated with wild-type MxA but not with the MxA T103A mutant**. A. HRPTECs were co-transfected with BKPyV TAg-expressing plasmid and FLAG-tagged wild-type MxA-expressing plasmid. Control transfections were the TAg-expressing or wild-type MxA-expressing plasmid alone. Immunoprecipitation assay was performed by immunoprecipitation with anti-SV40 TAg antibody. The TAg-binding MxA in the TAg-associated immunocomplexes was assessed by subsequent immunoblotting with an anti-FLAG antibody. The protein levels of inputted MxA, TAg, and GAPDH were determined through immunoblotting using their respective specific antibodies. B. HRPTECs were co-transfected with either FLAG-tagged MxA or FLAG-tagged MxA mutants (K83A and T103A)-expressing plasmids, along with the TAg-expressing plasmid. The immunoprecipitation assay was conducted using an anti-SV40 TAg antibody, followed by immunoblotting with an anti-FLAG antibody.

Because MxA is an important mediator of the IFNα-related antiviral response, and BKPyV TAg is a key early protein for viral replication [24], we first determined whether there was an interaction between MxA and TAg. HRPTECs were co-transfected with BKPyV TAg-expressing plasmid and FLAG-tagged wild-type MxA-expressing plasmid. Control transfections were the TAg-expressing or wild-type MxA-expressing plasmid alone. Immunoprecipitation assay showed that anti-TAg antibody pulled down MxA protein, as shown by subsequent immunoblotting with anti-FLAG antibody. This interaction was observed in the cell lysate extracted from cells co-transfected with both TAg-expressing and MxA-expressing plasmids but not in the lysate from cells transfected with TAg-expressing or MxA-expressing plasmid alone [Fig. 5A]. These findings confirmed MxA co-precipitated with TAg and indicated an interaction between these two proteins.

Given the observed loss of MxA-mediated suppression of BKPyV TAg and VP1 expressions in the T103A mutant-transfected cells but not the K83A mutant-transfected cells, we proceeded to investigate the interaction between TAg and these two mutants. Immunoprecipitation assay demonstrated co-precipitation of TAg with wild-type MxA and K83A mutant as anti-TAg antibody pulled down the wild-type MxA and K83A, evidenced by subsequent immunoblotting using anti-FLAG antibody [Fig. 5B]. In contrast, the expression of T103A mutant in the co-precipitated complex of TAg and MxA was reduced. These findings indicate that the amino acid position 103 on MxA plays a critical role in the interaction between TAg and MxA. Mutation at this site disrupts the interaction, potentially resulting in the loss of MxA's anti-BKPyV activity.

**Fig. 6:**
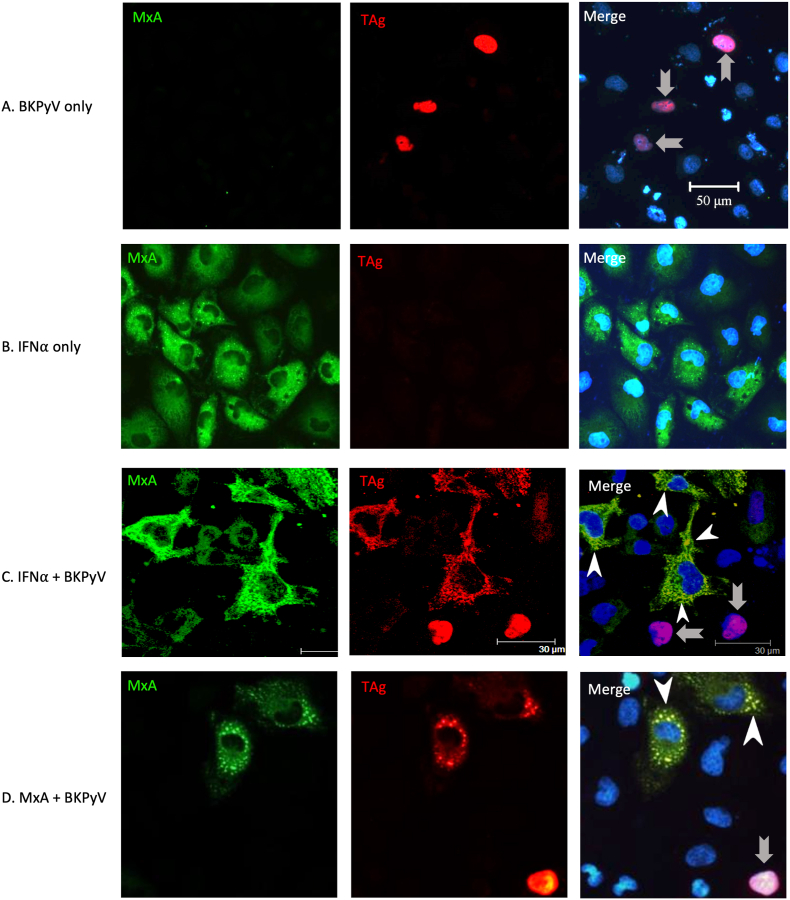
**MxA and BKPyV TAg are colocalized in the cytoplasm**. A. HRPTECs were infected with BKPyV (1 × 10^6^ copies/mL). After 72 h, cells were fixed and stained for MxA and TAg. B&C. HRPTECs were initially infected with BKPyV (1 × 10^6^ copies/mL) for 2 h, followed by incubation in the absence (B) or presence (C) of human IFNα (10 ng/mL) for an additional 72 h. D. HRPTECs were transfected with wild-type MxA-expressing plasmids for 12 h and infected with BKPyV (1 × 10^6^ copies/mL). After 72 h, cells were fixed and stained for MxA and TAg. MxA was labeled with anti-MxA and a green fluorescent (Alexa Fluor 488) secondary antibody, and BKV TAg was labeled with anti-SV40 TAg and a red fluorescent (Alexa Fluor 647) secondary antibody. In MxA-expressing cells, MxA exhibited colocalization with BKPyV TAg in the cytoplasm, displaying a diffuse granulate pattern (indicated by white arrowheads) (C&D). In BKPyV infected non-MxA expressing cells, TAg could be seen exclusively in the nucleus (indicated by grey arrows) (A, C&D).

On the basis of the protein-protein interaction between TAg and MxA, we conducted an immunocytochemistry analysis to localize TAg and MxA in cells. In BKPyV-infected HRPTECs, TAg staining was detected exclusively in the nucleus [Fig. 6A]. Upon IFNα stimulation, MxA staining could be seen in the cytoplasm of most cells [Fig. 6B]. In MxA-expressing cells, cytosolic MxA staining displayed a granulate pattern and colocalized with TAg. In contrast, intranucleus but not cytosolic staining of TAg could be seen in cells without MxA expression [Fig. 6C]. MxA transfected HRPTECs also showed similar results. Colocalized TAg and MxA couold be seen in the cytoplasm of MxA-expressing cells, and intranuclear staining of TAg could be only seen in MxA non-expressing cells.

## 4 Discussion

Type I interferons (IFNα/β), which are an integral part of our innate immunity and possess broad-spectrum antiviral properties, exhibit inhibitory effects on various DNA and RNA viruses, such as orthomyxoviruses, hepatitis B and C viruses, and coronaviruses [[25], [26], [27]]. Notably, our findings demonstrate the antiviral efficacy of IFNα against BKPyV.

Our data showed that both IFNα and its downstream product, MxA, were potent antiviral agents against BKPyV. Mouse IFNα also demonstrated antiviral effects against murine polyomavirus. This might give us a new option for treating patients with BKPyV infection. BKPyV infection after kidney transplant can be donor-derived or reactivation from the recipient. Early BKPyV infection is a risk factor for subsequent BKPyVAN. Since there are no satisfactory anti-BKPyV agents currently, it is worth conducting research on utilizing low-dose IFNα as a treatment option for recipients having BKPyV infection or BKPyVAN. More studies are needed before drawing conclusions.

The antiviral effects of IFN are carried by IFN-induced proteins encoded by ISGs. These ISGs include OAS, PKR, IFIT, and Mx. Human MxA, also known as Mx1, can block the early steps of the viral replication cycle [28]. Our data showed overexpressed MxA in HRTEPCs could inhibit BKPyV. Knocking down MxA expression in these cells could partially impair the anti-BKPyV effect of IFNα. The reason why there was only slightly reduced IFN antiviral activity after knocking down MxA might be that other ISGs still retained their antiviral function. Only knocking down MxA expression cannot totally abolish the antiviral effect of IFNα.

It has been confirmed that the antiviral effects of MxA against influenza viruses require oligomerization and the formation of a ring-like structure of MxA around viral nucleocapsids, which immobilizes them in the cytoplasm and blocks their function [29,30]. The BKPyV genome early region encodes TAg and tAg. The TAg contains a nuclear localization signal (NLS) in the N terminus, a DNA binding domain, and a helicase domain that regulates viral DNA replication and is primarily localized in the nucleus [31,32]. TAg can also recruit host DNA polymerase α for its own viral genome replication [33]. Upon BKPyV infection, the virion enters the host cell by caveolae-mediated endocytosis. BKPyV becomes uncoated in the endoplasmic reticulum and then enters the host nucleus. Viral early genes, including TAg, then transcribe in the nucleus and translate into the cytosol. TAg enters the nucleus and initiates viral DNA replication [34]. In our study, we have seen IFNα treatment reduced BKPyV viral load, and TAg colocalized with MxA in the cytoplasm in BKPyV-infected HRPTECs. We have also confirmed the existence of protein-protein interaction between MxA and BKPyV TAg by co-immunoprecipitation experiment. Taking these together, the antiviral effect of MxA against BKPyV is through trapping TAg in the cytoplasm and preventing its nuclear translocation, a crucial process for viral genome replication.

MxA has three domains: an N-terminal GTPase GTP binding domain that binds and hydrolyses GTP, a middle domain, and a C-terminal GTPase effector domain [18,23].

One study described mutations in the proximal motif of human MxA diminished antiviral activity against orthomyxoviruses but not bunyaviruses. Mutations in the distal motif of MxA abolished antiviral activity against both viruses. The distal sequence may serve a conserved structural function [35]. Another research studied the MxA antiviral activity against hepatitis B virus (HBV) showed the central interactive region of MxA is essential for MxA-HBcAg interaction but not the N- nor C-terminal of human MxA [36]. One study showed GTPase activity is not necessary for MxA to inhibit HBV replication [37].

These studies imply that different viruses interact with different domains of human MxA, and the importance of GTPase activity in antiviral effect is not universal.

To study which domain of MxA is important for its antiviral effect against BKPyV, we have created several MxA mutants. Lys-83 of MxA is a part of the phosphate-binding loop. K83A mutant abolished GTP binding [38]. T103A mutant substitutes a threonine to alanine at the N-terminal of MxA and abolishes its GTPase activities but not GTP binding [39], which diminishes MxA dimerization [40]. Our result showed that K83A retained its antiviral activities, but T103A lost its antiviral effect against BKPyV. Compared with K83A, T103A had diminished protein-protein interaction with TAg. Amino acid position 103 of MxA plays a crucial role in its antiviral properties against BKPyV related to its interaction with TAg.

In conclusion, our study demonstrates that both IFNα and its downstream protein, MxA, have potent antiviral effects against BKPyV. T103A is a dominant-negative mutant of MxA for its antiviral properties against BKPyV. Our findings suggest a potential new treatment option for BKPyV infection. Further studies are needed for the clinical application of IFNα in treating BKPyV infection.

## Figures and Tables

**Fig. S1:**
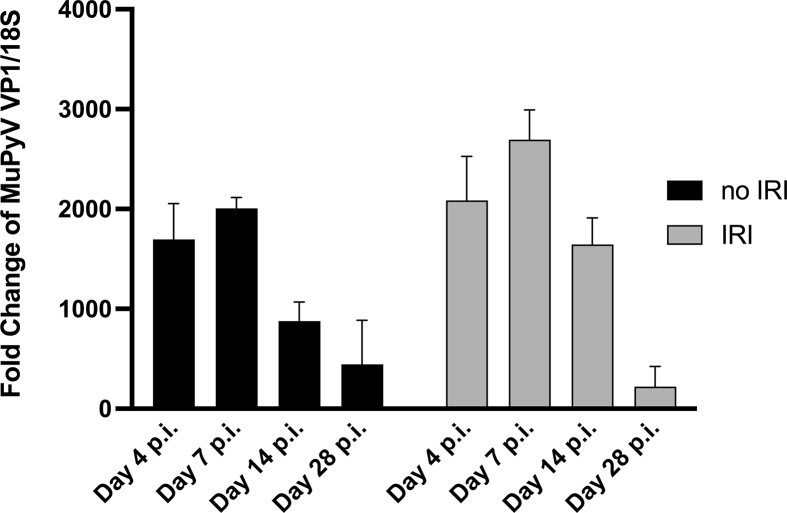
Eight-to ten-week Balb/c female mice received 30 min-ischemic reperfusion injury (IRI) of a unilateral kidney. Mice were then intraperitoneally inoculated with MuPyV wild-type strain (2 × 10^6^ plaque-forming units). Bar graph shows the fold change of mice kidneys MuPyV VP1 mRNA relative expression using 18S rRNA (18S) as reference at different time points post virus inoculation. Day 7 has the highest VP1 mRNA expression. Each experimental group comprised 4 to 6 animals. The results are presented as mean ± SEM.
